# Accounting for Material Reality in the Analytic Subject

**DOI:** 10.3390/bs3040619

**Published:** 2013-11-20

**Authors:** Robin McCoy Brooks

**Affiliations:** New School for Analytical Psychology, 927 N. Northlake Way, Suite 220, Seattle, WA 98103, USA; E-Mail: robin.mccoy@comcast.net; Tel.: +206-947-7078

**Keywords:** Carl Gustav Jung, subjectivity, science, Slavoj Žižek, Adrian Johnston, Jean Laplanche

## Abstract

Scientific advances made in the 21st century contend that the forces of nature and nurture work together through an ongoing series of complex correspondences between brain and mental activity in our daily activities with others. Jung’s cosmological model of the psyche minimizes the fundamental corporeal condition of human nature and as such is critiqued and amended, influenced by the transcendental materialist theories of subjectivity inspired by Žižek, Johnston and Laplanche.

## 1. Introduction

“It is not easy formulating a metaphysical position that meets the demands of a material world; there is still a lot of philosophical work to do.”

([1], p. 582)

Contemporary sciences deeply engage the role of the “sensuous brain” in emotional life, decentering long held psychoanalytic views of mind, brain, libidinal economy and subjectivity [2]. Various psychoanalytic traditions have opened up and are extending our understanding of the relational unconscious in psychoanalytic treatment (such as in transgenerational trauma). However, contemporary theory has not adequately articulated how material forces (bodily and environmental) influence, or impinge, upon the process of subjectification: the formation of the subject. A neuroscientific stance contends that the forces of nature and nurture work together through an ongoing series of complex correspondences between brain and mental activity in our daily activities with others [3]. New materialism theories do not confine nature to a singular biological body but extend materiality to the more-than-human. They view the interface of history, culture, technology, political and scientific environmental practices to be as equally valid as social constructions in the ways we can account for ourselves as subjects [4]. Thus, as human beings we are living and dying in the midst of an agentic natural world whose actions have consequences for both human and non-human alike. In this view, we are always already engaged in multiple agentic-often invisible- ecological/and biological materialisms (such as air and water pollution, epigenetics) whether we know it or not [5]. 

How the body gives rise to the mind certainly perplexed Jung as he wondered “how life produces complex organic systems from the organic” in his final reformulation of the psychoid archetype in 1946 ([6], para. 375). He was of course limited in his view (as we are) due to limits of the knowledge systems (political, social, scientific, cultural, philosophical *etc.*) that he was historically entangled in. In this paper, I critique aspects of Jung’s model of subject formation because, in my opinion he minimizes the fundamental material condition of human nature by privileging independent transcendent sources of subjectivity that originate *outside* of personal experience and personal unconscious fantasy. While retaining Jung’s crucial post-Kantian insight of a split subject I attempt to articulate a model of subjectivity that recognizes the correspondences between the social/biological/ and psychical realms. To that end, I turn to a transcendental materialist theory of subjectivity inspired by post-Lacanian psychoanalysts/philosophers such as Slavoj Žižek, Adrian Johnston and Jean Laplanche. These theorists view the psychoanalytic subject through unique materialist/metaphysical lenses.

## 2. Debates in Science and Psychoanalysis

An explosion of knowledge about the neurodynamics of the brain is stimulating many kinds of cross disciplinary applications. This is notable in the work of John Bowlby (attachment theory), Antonio Damasio (neuro-biological model of consciousness [7]), Mark Solms and Oliver Turnbull (neural unconscious paradigm [8]), Christian Roesler (epigenetic conceptualization of archetypes [9]), Julia Kristeva (depression [10]) and Johnston and Malabou [2]. These authors creatively rethink subjectivity through the lenses of neuro-biology, philosophy and psychoanalysis, thereby opening up new conceptual possibilities. 

How can psychoanalysis remain culturally relevant in an age when neuro-cognitive science seems to be emerging as the dominating master discourse? One stance is for psychoanalysis to *do nothing* holding the view that the analytic method already is a ‘science’ that is sufficient onto itself that will stand the test of time on its own merits. Proponents of this stance (although richly varied) generally assert that neuro-science is irrelevant to the way practice and to our understanding of the human subject [11,12]. In the much discussed series of papers, Blass and Carmeli make the case against both neuro-science and neuropsychoanalysis by calling into question the claim that neuroscientific findings are relevant for the justification of psychoanalytic theory and practice [13,14]. They argue for the efficacy of the analytic stance which in their minds focuses on “the understanding of meanings and the role of interpersonal discourse in discerning and justifying these meanings”, *versus* what they call “biologism” which asserts “only what is biological is real” ([15], p. 1584). A case could here be made that adhering to psychoanalytic principles alone as the complete means for understanding the mind and its self-disruptions (to include brain disorders) can be a reverse variation of biologism, a kind of psychoanalytic-ogism or psychoanalytic reductionism. 

Another response is for psychoanalysis to creatively assimilate neuro-science by entering meaningful correspondences between brain and mind. Assimilation of material from one discipline to another can take on various and discrete distinctions that Talvitie and Ihanus [15] organize into three neuropsychoanalytic conceptions that I will amend for our purposes here.

The reductionist conception reduces the basic assumptions of one theory of mind or brain to the other. However some authors, who identify themselves as “new wave reductionists” (*versus* classical) clarify their position as one that while interested in mechanistic explanations, does not view the autonomy of psychology and reduction as contradictory views. They favor “explanatory pluralism…a non-reductivist approach that is neither reductive nor anti-reductive” ([15], p. 1585). A less favorable variation of this stance might see it as psychoanalysis subordinating itself to the leading science, like Anna Freud’s “identification with the aggressor” [16]. From this perspective, one would interpret psychoanalytic questions through the neuroscientific lens as if neuroscience is the basis of all human sciences, in a kind of pervasive neuro-ism [11]. 

A second or *hybrid* approach attempts to integrate (*versus* reduce) or unify the views of psychoanalysis and neuroscience without negating the basic assumptions of either [15]. While this may be a more diplomatic strategy it can minimize or whitewash the irreducible aspects of either discipline, ultimately diminishing the potency of both the organic and immaterial (*das ding an sich*) views. 

Allan Schore’s (the “American Bowlby”) integration of human development and neuroscience culminating in his attachment theory model is an example of a stance which seems to lose the depth dimension of psychoanalytic reflection [17]. There are also many prejudiced notions on the psychoanalytic side. For instance, many contemporary Anglo-American psychoanalysts have theoretical biases in their collective conviction that infants are primarily object seeking [18,19]. This turn in conceptual framework was emboldened by the unification of neuro-scientific models of attachment theory; turning people from the centrality of Freudian psycho-sexuality drive theory [20]. In my opinion, this loses the richness of alterity, of the human psyche in its excesses of desire, its aberrant drives and intrapsychic conflicts. 

Talvitie and Ihanus’ third approach or an *interfield* conception *does not* attempt to unify any theories but respects the gap between the natural sciences and the humanities [21]. Roesler is an example of an interfield theorist who argues for an epigenetic model of the archetypes, viewing Jung’s own biological arguments as outdated [8]. While agreeing with Knox [22] and Hogenson [23]—also inter-field theorists—that the emergence model is so self-evident as to be ‘banal’, he challenges their contentions that archetypes have a universal component (*a priori*). Malabou and Johnston, both philosophers—the latter a psychoanalyst—compellingly embrace neurobiology. They acknowledge both the irreducible aspects of each discipline to each other (of mind and brain). In a sustained dialogue with the disciplines of philosophy, psychoanalysis and neuroscience they radically rethink what it means to be a human subject. 

The French psychoanalytic tradition (in general contrast to many American models), particularly the work of Jean Laplanche continues to emphasize the centrality of infantile sexuality. He advances and clarifies Freud’s central ideas of drive and instinct and the interplay between what is physiological (biological) and what comes from outside of the infant via (enigmatic/ unconscious) messages from the (m) other. In other words, sexuality is not endogenous to the infant but emerges from the fundamental asymmetrical relationship (attachment) with the other due to its dependence (*hilflösigkeit*) and difference [24]. Although Laplanche does not rely on neuro-scientific developments to broaden his theories, his theories “lean upon” (“*anlehnung*”) the biological but cannot be reduced to it [25].

## 3. Jung and Kant

It is well known that Jung was closely aligned to and influenced by threads of thinking derived from Kant’s transcendentalism, generally associated with the philosophical movement known as German Idealism. Kant’s subject was split between two irreducible realms-the first positing a pure noumenal, transcendental a-temporal, logical, “I” and the second positing a phenomenal, egoistic, spatio-temporal “I”. The pure “I” can never know itself as *arche* (ipseity), and is inaccessible as an object of experience. Therefore, Kant stated; “through inner experience I always know myself only as I *appear* to myself” ([26], pp. 26–27, my italics). In other words, the “phenomenal I” intuits and does not cognize a *thing in itself* and what is presented through a sense is always an *appearance* ([27], p. 190). Yet, it is consciousness that turns all “presentations” (*Vorstellung*) into thoughts, even though one is not able to adequately cognize the conditions that make the act of cognition possible [27]. *Vorstellung* can be translated as meaning whatever is given as present to awareness and within that Kant designated various kinds of general appearances ([27], p. 155). The term *Vorstellung* was mistranslated into the English editions of Jung’s collected works calling immediate experience “image” or “idea” rather than “appearance” or “phenomena” therefore rendering *Idee* (idea) and *Vorstellung* (representation and presentation) as the same ([28], pp. 8–11). 

Returning to the specific topic of subjectivity, Johnston succinctly summarizes the Kantian arrangement of the divided subject thus:

Self-consciousness is limited to apprehending the phenomenal subject. Midway between intuition and reason, the understanding necessitates an iterable “I” accompanying every determinate act of cognition…From this iterability, reason, in accordance with the transcendental idea of psychological unity of subjectivity, treat the iterable “I” of the understanding as indicative of the need for a regulative principle of a timeless self-sameness. This is nothing other than the purely noumenal subject, a subject that must be posited given the systematic aspirations of the interests of reason, although no experiential correlate can ever be adequate to this idea

([29], pp. 103–104)

Johnston’s characterization of a zone “midway between intuition and reason” is an elaboration of Kant’s boundary concept. Kant introduced what he called and empty space’ or gap between the two concepts, which was neither purely noumenal (negative) nor phenomena (positive), or jointly both ([30], pp. 4, 354; [31], p. 497). In another context, Eyal’s Weizman’s depiction of a fixed border that separates deeply fragmented, constantly shifting and elastic [sovereign] territories’ while somehow benefiting both realms comes to mind ([32], p. 7). In this context, Weizman is describing his view of the wall separating Palestinians from Palestine.

It cannot be understated how essential Jung’s use of Kant’s boundary concept was to his project; indeed he attributed his philosophical epistemological basis of *esse in anima* (soul) to Kant ([31]; [33], p. 123). For Jung, human nature was divided between the inconsistent surfaces of the phenomenal realm that was contained by a unifying transcendent ground beneath its surface. Jung then situated a “world soul”, *anima mundi*, or “spirit of God” in the very heart of the noumenal territory—whose explicit goal was to advance the individual (and the whole of mankind) towards a union of spirit and soul (“*unio mentalis*”) in the body ([34], para. 707; [35], paras. 388, 293). The archetypes, or emmisarial productions of the world soul, could breach the irreducible boundary between the realms and make themselves known through phenomena (affect, instincts) and representation (image, idea). The self therefore *was* a “borderline concept,” a mediating and unifying force located in the gap between the phenomenal and noumenal realms, yet containing both ([36], p. 258). 

Jung was not interested in personal unconscious processes, which he equated with Freud’s unconscious considering its function as inferior to the objective psyche [37]. Understanding human experience therefore was largely dependent on the interpretation of archetypal “images”, which of course could not be understood in terms of merely personal unconscious processes [38]. This view of subjectivity does not account for sources of signification deriving from personal unconscious processes (fantasies, dream material, transference phenomena, *nachträglich* shifts in temporality, *etc.*), or those that emerge in relationship, or from relation to organicity (affect, brain states, physical suffering, the role of drives and instincts and organic and psychic trauma). 

## 4. The Metaphysical Subject in a Material World: Žižek, Levinas, Laplanche

Central to Žižek’s project is the “redeployment of a German idealist theory of subjectivity” as it is revised through the lens of post-Lacanian psychoanalytic metapsychology ([39], p. 125). While Jung and Lacan’s projects are epistemologically incompatible, Žižek and Jung both found abundant theoretical inspiration in German Idealist theory. Žižek’s contemporary re-reading of German Idealist theory blows fresh air into the possibilities of reviving aspects of Jung’s model of subjectivity through a Žižekian lens. In what follows, I roughly sketch relevant aspects of Žižek’s arguments while reworking aspects of Jung’s model of subjectivity that crisscross and overlap this discussion within other sections along the way.

Most compelling of Žižek’s claims is his assertion that the Cartesian conception of a split subject as Kant, Schelling and Hegel rethink it is not only relevant today but also central to his project (one that has spanned for decades). Johnston succinctly summarizes Žižek’s central claims (as derived by late modern transcendental philosophy) thus:

The subject is “emergent in relation to the body-that is to say, such ‘immaterial’ (or more accurately, more-than-material) subjectivity immanently arises out of a material ground…Cogito-like subjectivity ontogenetically emerges out of an originally corporeal condition as its anterior ground, although, once generated, this sort of subjectivity thereafter remains irreducible to its material sources…subject conditions immanently arise out of a series of conflicts and tensions internal to the foundational embodied condition of human nature, a nature inherently destined for denaturalization”

([40], p. 231)

Žižek’s basic thesis opens up psychoanalytical consideration for the interpretation of both unconscious *mental* and *organic* influences to selfhood. Hence, he creates a space for investigation into the aspects of the corporeal constitution of human nature that in addition to the human sciences includes the cognitive, neural and molecular dynamics (to include epigenetics) at play in becoming a person. While retaining the assumption that “truth” emerges from encounters with the Lacanian Real (somewhat akin to Kant’s pure negativity) and is therefore the major source of human knowledge, cogito-like subjectivity emerges out of its corporeal condition and is subject to tensions internal to its foundational embodied condition ([40], p. 230). These tensions may be in response to productions of the mind and/or the emotional brain as well as external sources. In contrast, c*ogito*-like subjectivity for Jung emerges instead from the productions of a world soul located outside of the corporeal individual and is transported via the archetypes through the psychic matrix of the collective unconscious penetrating the body via the psychoid spectrum through affect, instinct or representation (image/idea). 

Žižek makes a crucial distinction between *subjectivity* and *subjectification.* He defines the *self or subject* as pure negativity (the noumenal “I”) such as the Kantian subject-as-Thing or Cartesian cogito. The subject is *not* the “I” of the “self” that is associated with mental processes or the egoistic phenomenal “I” ([39], pp.166–167). *Subjectification* as clarified by Johnston, is defined “as a series of interminable efforts, of vain attempts structurally doomed to partial success at best…to reinscribe the subject within gentrified domain of actualized re/presentations” ([39], p. 167). Subjectification therefore is an anxiety-ridden and frenzied attempt to re-absorb the void revealed by the negative rupture of what is alien to the phenomenal “I” back within the order of the ontological plane from which it broke [39]. Put another way, the phenomenal I takes an inner distance from its -self (the noumenal “I”) to absorb the trauma of the void made known to itself through alterity’s rupture. On this Žižek’s comments:

“We could say, paradoxically, that the subject is substance [by substance is referring to the stuff of ‘the thing’] precisely in external, positive Entity, existing in itself: “subject is nothing but the name for this inner distance of ‘substance’ towards itself, the name for this empty place from which the substance can perceive itself as something ‘alien’. Without this self-fissure of the essence, there can be no place distinguished from essence in which essence can appear only in so far as it is already external to itself”

([41], p. 167)

From this perspective, subject will always be “external to itself” while existing “in itself”-an ongoing oscillation between alteration and iteration (25). Žižek’s exquisite extension of Kant’s boundary concept can apply to the basic elements of Jung’s model of subjectivity with the crucial exception of the sources of tension that purportedly ignite the mind’s emergence. Žižek accounts for subjectivization’s genesis (or the body giving rise to the mind) in two ways—“something going terribly wrong”, or an ontogenetic account—“a kind of snag in the biological weave” ([42], p. 59). Zizek is extending his reading of the Lacanian Real to include both material and psychic sources of traumatizing sources of tension that continually rupture and make our ontological incompleteness known. Jung, in contrast held the objective psyche as the foundational ground and central source of ultimate truth via the founding principles of being that he described later as the psychoid realm. Jung’s subject was constructed through inter-actions with psychoid entities that were guided through the emanations of a divine world soul.

Something goes “terribly wrong” in Žižek’s ontology when a person encounters what Lacan called the “Real”. Over sixty years ago, Lacan claimed he was returning to what he perceived was an often abandoned insight in psychoanalysis about the intrinsic unintelligibility of the unconscious [43]. The goal of analytic treatment, he claimed was not to elevate ego functions *vis-a-vis* the unconscious but contrarily to confront the barriers to experiencing the “Real” or the site of the “traumatic truth” [43]. Žižek rearticulates (from Lacan through a Kantian lens) the Real as “voids entirely immanent to the representational fabric of reality rather than presupposed as a pre-representational transcendence” ([41], p. 173)

The Real described in this way is somewhat akin to the gap between Jung’s collective unconscious and person that he deemed the “psychoid”. It is in this gap or boundary that the possibility for subjectification could occur via Jung’s self (as an archetype), through its implosion into the body and the individual’s response to it. The mechanism that regulates the possibility of Jungian individuation is personified or reified into a single agent from beyond (God, world soul), in contrast to the “Big Other”, of our cultural thrownness in Lacanian parlance. 

Žižek’s subjectification is evoked through encounters with the Real (via the many appearances of the virtual character of the Big Other that appear in various materialisms such as politics, economics *etc.*). The Real in its various dimensions is a dynamic process embedded in our everyday codes of behavior (conscripted socially amongst relations with people, one’s self, things) and revealed in the cracks (gaps or inconsistencies) of the virtual symbolic matrix that structures reality for us. Including the materialist sensibilities of Alaimo [4] noted in the introduction, I include the conscription of material agencies as well as the psychical in the various realms of the Real such as political, economic, technical, and scientific systems of coding as well. These gaps or inconsistencies can be partially detected in their traces via the effects of our fantasies, our dreams, or other evidences of the inscription of the subject in its field of objects (metaphor, metonym, *après coup*, *etc.*) or the counter inscription of the Other onto the subject. We are, in this model always already embedded in this social symbolic/material (I add) matrix, and “subjective symbolic identity” is historically determined [43]. Zizek makes a compelling partial parallel between the Lacanian virtual symbolic order that structures reality for us and the controlled virtual reality portrayed in the tri-part movie the “Matrix”. He relays a poignant shard of dialogue between Morpheus (the leader of the resistance group) and Neo, a naive and future savior figure:

MORPHEUS: “It’s that feeling that you have had all your life. That feeling that something was wrong with the world. You don’t know what it is but it’s there, like a splinter in your mind, driving you mad…The Matrix is everywhere, it’s all around us, here even in this room…it is the world that has been pulled over your eyes to blind you from the truth. NEO: What truth? MORPHEUS: That you are a slave, Neo. That you, like everyone else, was born into bondage…kept inside a prison that you cannot smell. Taste, or touch. A prison of your mind”

[44]

Thus, from the beginning, the subject is divided, decentered and incoherently unintelligible to itself and subsumed in the groundless ground of the multiple dimensions of the Real. Analysis for Lacan can only be an ethical undertaking as its central task is an awakening to the truth of our desire that is barred by the unconscious fantasy that controls us.

The Lacanian subject encounters the Real through the Aristotelian notion of *tuché* (a unexpected fist in the gut) that is literally reinterpreted as a missed encounter with the Real. Similarly, Levinas thought of the subject *as* trauma, or of ethics (the site of the ethical relation to alterity) *as* traumatology ([45], pp. 90–94). He poignantly elaborated on the affective response of the body (trauma) to the effect of being taken hostage by the other’s demand [46] as being the heteronomous site of subject formation and ethics. The thought of an act can only be born through the violent and traumatic struggle of being overcome by the other’s demand. Contra Jung, no teleological account is sufficient for this primal ethical awareness [38]. 

Lacan links the *tuché* with the Real by arguing that such encounters radically destabilize ipseity and are therefore foundational to subject formation. The fundamental opacity of the other’s desire can utterly rupture the ego’s ability to assimilate or restore a sense of equilibrium. Lacan held that subjects’ manner of being could be transformed by investigating the questions posed at the traumatic sites ‘of truth’ imposed through the missed encounters with the Real [47]. 

The body gives rise to the mind by fielding its impingements in a kind of desperate attempt to restore libidinal equilibrium. These unpredictable and inassimilable disruptions to the helpless phenomenal “I” are constitutive to subjectivity and to one’s relationship to others. Again, Johnston artfully summarizes this dynamic process:

This shift of substance becoming subject is followed by subjectification processes in which certain master elements engage in the labor of hegemonic articulation, retroactively reconfiguring the very ground of being-substance from which they arose. This shift occurs both at the ontogenetic and phylogenetic levels

([39], pp. 173-174)

This statement clearly links the biological evolutionary influences to subject formation holding the mind in relation to its biology (or “a snag in the biological weave”) while not being reduced to it. Thus, subject (transcendent “I”) transcends corporeality (corporeal embedded phenomenal “I”) and in the Levinasian sense becomes “otherwise than” or “beyond being” [48]. Both Žižek, Johnston and Malabou (in separate works) attempt to address the mind and brain in relation to its psychic and neuro-unconscious aspects and their mutually constituting generativity [2,49].

Johnston correlates the emotion from a neurobiological perspective on emotion with drive. Dialoguing with the thinking of neuroscientist Damasio, and Žižek’s reading of Damasio, Johnston ‘re-elaborates’ cerebral organization, psychical apparatus and unconscious processes. He situates the neurobiological emotional brain within a paradoxical emotional libidinal economy—one which recognizes that emotions organize and coordinate cerebral activity. He states…“In the brain, there are no regulatory mechanisms of adaption to the external world and the environment without emotional adaption to the inside of the brain by the brain itself” (*i.e.*, the brain taking the drives and energetic tensions upon itself alone—my clarification) ([2], pp. 217, 219). He appropriates Damasio’s conception of a “protoself” (the primitive form of identity or cerebral unconscious) as a kind of central command post of auto-regulation between internal processes and the external world. He states:

The proto-self is a coherent collection of neural patterns which map, moment by moment, the state of psychical structure of the organism in its many dimensions [in a constant synthesis of different states of relation between body and psyche, as an equilibrium, in a word of the organism]

([2], p. 220)

This kind of organic self-regulatory nexus is perversely reminiscent of Jung’s diametrically oppositional concept of *anima mundi*, or world soul. Jung’s regulatory nexus or world soul is psychical, and embedded in a cosmic nether-land. 

Catherine Malabou in her work exploring how brain damage changes subjectivity contends that while the brain is sculpted by the contents of mental experience (of the self and its experiences), so do the brains affectations (such as in auto-affection for example) shape the subject’s experience of itself ([2], p. 46). The cerebral self is anonymous and a kind of primal core of corporeality, yet one that is implicitly an internal reminder of our mortality as the organism is always on the edge of impending partial or total collapse ([2], p. 223). This notion can shed new light on Freud’s ever haunting presence of a death drive ([2], p. 223). In contrast, the psychoanalytical unconscious, or Jung’s objective psyche does not believe in its own death representing only atemporal dimensions of experience. Malabou and Johnston contend that the link between the subject (personal unconscious) and the brain (proto-self) can best be theorized through affect. Consequently, the brain gives rise to the mind through the interplay of affective signification, the body’s autoregulation of it and the process of subjectivation as outlined above.

## 5. Laplanche

Laplanche’s insight into the central role of the “sexual” unconscious is relevant to the theoretical discussion at hand and gives clinical flesh to Zizek’s transcendental materialist theory of subjectivity. Indeed, he considered himself to be a materialist and stated: “I think that anything that exists in the realm of the mind also exists somewhere in space, in the brain” [50]. In the same vein, he later stated: “I have never left the body and I have never opposed the body to the mind. By placing the drive and instinct in opposition I am not opposing the psychical to the somatic” ([24], p. 11). He enlarges the Freudian notion of infant sexuality into a “polymorphous perverse” infantile sexuality that can be translated into English as “the sexual”. Infant sexuality therefore is associated with the unconscious residue of the symbolization-repression and fantasy that is fundamental to the primal relationship with the “Big Other” from birth and is the object of psychoanalysis ([24], pp. 159–190). Laplanche takes the Lacanian Real and particularizes it to the asymmetric relationship between Big Other and infant. He argues that sexuality, broadly speaking, consists of universal unassimable communications from the mother to infant that are implanted in the infant’s body as “primal repressions”.

Self-preservation is also active in the infant from the start and mediates communications that are more reciprocal and less enigmatic. That is what is commonly understood as “attachment” (or adaptional) in today’s psychology. Laplanche contends that Freud anticipated what we know as attachment theory with his notion of “affection”. Thus, he states: “owing to the hegemony of attachment theory there is a risk that the debate over attachment and sexuality may never in fact take place, unless attachment can be accommodated within the framework of a rigorous metapsychology” ([24], p. 36). On the other hand, enigmatic (and untranslatable) messages that originate beyond language remain unmetabalized and untranslatable. These enigmatic elements eventually form a core “internal foreign body”—a sort of “alien inside of me, put inside me by an alien” ([51], p. 65). These he calls ‘source objects’ equivalent to drives that stimulate endless fantasy, both destructive and creative, throughout life. Laplanche asks us why the poet poetizes… except as a response to an enigmatic presence in an unknown future ([51], p. 223).

Laplanche views instincts as such to be self-preservative, adaptive and innately programmed to secure survival and homeostasis. The *sexual instinct* he claims is situated in the biological maturation of the organism and does not emerge until the pre-pubertal period. The *sexual drive* in contrast, is ubiquitously situated in infancy as Freud foresaw, is *not* innate, is connected to fantasy, and is necessarily repressed (and therefore unconscious). The abject helplessness (*Hilflössigkeit*) of the infant requires a helping other to compensate for the infant’s deficiencies of self-preservation and ensures that “the sexual” takes over. Thus, *the sexual order always already overlies the instinctual order* [52]. Unconscious sexual messages are exchanged from the mother to infant within the attachment relationship that remain unintelligible and “implanted” later appearing as what Laplanche refers to as “enigmatic signifiers” that are provoked through the transference in life and/or analysis where they can be detranslated, and retranslated [53]. In other words, the emergence of repressed fantasies (via enigmatic signifiers) in the analysis can unveil originary drive (part instinct to survive and part drive to reduce the recurring anxiety-ridden need), but the enigmatic core, like the Lacanian Real, is never “resolved” or “integrated”. Crucial to the Laplanchian analytical stance is the analyst’s capacity to maintain ‘the dimension of interior alterity which allows alterity to be set up in the transference, and for the reactivation and working through the originary enigmas ([50], pp. 228–229). That is, it is crucial that the analyst be in touch with his/her own enigma. 

Jung, (using Soni Shamdasani’s translation) conflated the terms drive and instinct in his final reconceptualization of what he would later refer to as the “psychoid archetype” ([35]; [54], pp. 258–260). Drives/instincts had two aspects in that they were dynamic instinctual patterns in human biology and secondly would enter into consciousness as images. Jung called them “*apriori* instinct-types”, or “instinct image[s]” stating that the image represent[ed] the *meaning* of the instinct’ ([35], paras. 398–399). Thus the drive/instinct was both psychical and biological and the instinct-*Vorstellung* could be translated into meaning through Jung’s method of active imagination. While both Jung and Laplanche viewed the psychical to always already overlie the biological order with regard to subject genesis, Laplanche’s elaboration is more profound on multiple levels. To begin with, Laplanche includes both psychical (sexual drive) and biological (self-preservation, attachment) relational elements from the beginning of life that shape whom and how we become and understand ourselves to the degree one ever can. He clearly distinguishes a nuanced model of drive and instinct and their correspondences in relation to infant/m-other from the beginning while accounting for a split subjectivity that is generated from these early relations between infant and world. The big Other for Jung is the world soul, and its correspondences via the archetypes with an isolated mind. Biological participation, for Jung in subject formation, is grossly understated and undeveloped in an already troubled concept. 

Central to Laplanche’s general theory of seduction is the concept of afterwardness (*après-coup* -Lacan, or *nachträglichkeit-* Freud). In a surprising homage to Jung, Laplanche cites both Freud’s concept of *Nachtraglichkeit* and Jung’s concept of retrospective fantasizing (*Zurückphantasieren*) into his own conception. He differs from both Jung and Freud as well as from Lacan’s earlier nod to Freud [51]. Freud proffered a determinist conception (what happens before determines what happens after) of *nachträglichkeit* which followed a temporal trajectory of trauma from past to future by positing that trauma implanted in the past can be reactivated later, although reinterpreted from the standpoint of a more sexual mature individual. Indeed, he discussed how that could increase trauma because of the added dimensions of meaning. Jung’s retrospective or hermeneutic conception involved a reinterpretation of experiences relating to past trauma through the lens of the archetype. That is, the actual experience of the past was devalued. Laplanche essentially adds two crucial elements in his extended version of *après-coup*; his translation model (identifying the trace of retroactive enigmatic translation, retranslation and reinterpretation) and the introduction of the relational unconscious through the (m) other infant relationship. 

Jung’s aversion to Freud’s sexual libidinal theory is well known as well as his dislike of reduction to the past. He was opposed to child analysis and did not develop a theory of childhood development. Fordham recalls attempting to discuss child therapy at a dinner party with the Jung’s. Of Jung he stated: “He was starting on a monologue when Mrs. Jung intervened: ‘You know very well that you are not interested in people, but [only] your theory of the collective unconscious’” ([55], p. 109). Jung clinically interpreted childhood motifs (to include mother symbology) as representing archetypes without considering the importance of actual childhood experiences or the fantasies of these experiences as having analytic relevance to subject formation [56,57]. Elsewhere, I elaborated on the rigid stance of epistemological authority that Jung relied upon when it came to the archetypal explications of the patient’s experience that can be noted in his method of amplification [38]. I state that the term “amplification” itself was a misnomer, in that it implied that Jung’s intent was to expand the signification of unconscious content, yet this process was in fact only a precursor to a formulaic reduction of the expanded material to a presumed archetypal core ([38], p. 87). His phenomenological-descriptive approach however, was one he retained for working with the personal unconscious and was derived from his earlier research with the word association test ([56], para. 174). This approach employed a discursive process between patient and analyst that expanded or opened up possibilities by following the patient’s own associations. Jung would probably be opposed to Laplanche’s notion of enigmatic signification entirely and his general theory of seduction. However, because Jung minimized or did not understand the importance of personal unconscious processes in general most particularly in childhood development or subject formation, this gap in his theory and practice, to my mind requires serious supplementation. Laplanche’s work revives Freud’s abandoned theory of seduction and extends it to embrace a metapsychological position that meets the demands of a corporeal reality across the arch of an individual’s life. Laplanche’s model can be incorporated into the clinical realm that recognizes neural diversity and other materialisms as relevant to subject formation, and adds crucial psychoanalytic dimension to personal unconscious processes as well.

## 6. Concluding Discussion: Returning Jung’s Subject to the Material World

Via Žižek, Johnston and Laplanche I have described a process of subjectification that leans on the biological yet is not reduced to it. The forces of nature (brain/body, the biologic) and nurture (mind, the psychoanalytic) work together through an ongoing series of complex correspondences between brain and mental activity. These forces interface with co-extensive material agencies (m (other), political, economic, technological, social environments, *etc.*) from the beginning of human life. It is beyond the scope of this paper to elaborate further on what and how these coextensive material agencies interact in subjectification other than the scope of the brain and the mind, an unfortunate omission and certain topic for another essay. From the beginning, the subject is divided, decentered and incoherently unintelligible to itself, subsumed in the groundless ground of the multiple dimensions of the Real. For Laplanche, a primal psychic split occurs simultaneously with the birth of the ego and the repressed unconscious because of infants failed attempts to translate the m (other’s) unconscious sexual messages despite the adequacy (or inadequacy) of the attachment relation ([52], p. 12). The instinctual realm (that regulates self-preservation), in other words cannot adequately regulate this surplus of demand unconsciously initiated from the (m) other, or her environments.

Johnston correlates emotion with a neurobiological drive in brain/mind collaboration between cerebral organization, psychical apparatus and unconscious processes. In Johnston’s perspective, emotions organize and coordinate cerebral activity in an adaptive (self preservational) stance to the environment. The psychical stuff of the thing (or noumenal “I”) appears to have a material counterpart in the brain’s cerebral unconscious (adapted from Damasio’s conception for the “protoself”), the central command post of auto-regulation between internal processes and the world. In both theories, the body and mind are co-extensive agents to the rise of the immaterial human subject from its corporeal origins. 

Jung’s notion of the subject does not allow for new or unthought configurations of alterity that can account for present experience within personal unconscious processes, or co-extensive material systems described above. The mechanism that regulates the possibility of Jungian individuation is personified or reified into a single agent from beyond (God, world soul), in contrast to the “Big virtual Other” of our cultural thrownness in Lacanian parlance. Following Žižek’s reading of Kant, I retain crucial aspects of Jungian transcendental subjectivity while reconceptualizing its source of genertivity from the objective psyche to *alterity.* Encounters with alterity are instead initiated through the individual’s uncanny encounter with the “other’” located in the gaps of a groundless extraontological reality at the organic and psychical enigmatic core of the human condition. This enigmatic core of the subject was designated by Kant as the “noumenal I”, by Jung as the self (implanted via the self-archetype via the world soul), and Žižek as “subject”. For Žižek and Laplanche, the subject is always “external to itself” while also existing “in itself” or emergent in relation to its material ground (the body). 

Jung’s self is an archetype generated from an external cosmological nexus whose only contact with corporeality is through the instincts or affect. Unlike alien enigmatic signification generated in the m (other) in relation with her infant in the Laplanchian landscape, Jungian archetypes arrive from inner or outer space (a cosmic other) like meteors crashing into the earth. Staying with Žižek’s model, I demote the archetype from its cosmologic origins *to* referents, representations or enigmatic signifiers that *emerge in personal unconscious processes (affect, language, sensation and fantasy).* This includes the virtual symbolic matrix (codes provided by culture) that interfaces with the body/mind *and* is coextensive with the body in the material world and its many environments. While some Jungian theorists are attempting to scientize the archetype as a viable representative of the collective unconscious, Jung’s lingering totalizing cosmology remains crucially problematic.

## References

[1] Hogan T. (1993). From Supervenience to Superdupervenience: Meeting the Demands of a Material World. Mind.

[2] Johnston A., Malabou C. (2013). Self and Emotional Life Philosophy, Psychoanalysis and Neuroscience.

[3] Aamodt S., Wang S. (2011). Welcome to Your Child’s Brain.

[4] Alaimo S. (2010). Bodily Natures Science, Environment, and the Material Self.

[5] Alaimo S., Hekman S. (2008). Material Feminisms.

[6] Jung C.G. (1970). The Conjunction. The Collected Works of C. G. Jung.

[7] Damasio A. (1999). The Feeling of What Happens.

[8] Solms M., Turnbull O. (2002). The Brain and the Inner World: An Introduction to the Neuroscience of Subjective Experience.

[9] Roesler C. (2012). Are archetypes transmitted more by culture than biology? Questions arising from conceptualizations of the archetype. J. Anal. Psychol..

[10] Kristeva J. (1989). Black Sun Depression and Melancholia.

[11] Brothers L. (2002). The trouble with neurobiological explanations of mind. Psychoanal. Inq..

[12] Verhaeghe P. (2012). Psychoanalysis in Times of Science. Journal of the Jan van Eyke Circle for Lacanian Ideology Critique.

[13] Blass R., Carmeli Z. (2007). The Case against Neuropsychoanalysis: On Fallacies Underlying Psychoanalysis’ latest Scientific Trend and Its Negative Impact on Psychoanalytic Discourse. Int. J. Psychoanal..

[14] Carmeli Z., Blass R. (2013). The case against neuroplastic analysis: A further illustration of the irrelevance of neuroscience to psychoanalysis through a critique of Doidge’s *The Brain that Changes Itself*. Int. J. Psychoanal..

[15] Talvitie T., Ihanus J. (2011). On neuropsychoanalytic metaphysics. Int. J. Psychoanal..

[16] Freud A. (1993). The Ego and the Mechanisms of Defense.

[17] Allan Schore. http://www.alanschore.com/.

[18] Fairbairne R. (1952). Psychological Studies of the Personality.

[19] Waintrater R. (2012). Intersubjectivity and French Psychoanalysis: A Misunderstanding?. Stud. Gender Sex..

[20] Fonagy P. (2001). Attachment Theory and Psychoanalysis.

[21] Darden L., Maull N. (1977). Interfield theories. Philos. Sci..

[22] Knox J. (2003). Archetype, Attachment, Analysis. Jungian Psychology and the Emergent Mind.

[23] Hogenson G.B., Cambray J., Carter L. (2004). Archetypes: Emergence and the Psyche’s Deep Structure. Analytical Psychology: Contemporary Perspectives in Jungian Psychology.

[24] Laplanche J. (2011). Freud and the Sexual: Essays 2000–2006.

[25] Hinton L. (2013). New School for Analytical Psychology.

[26] Kant I., Dowdell V.L. (1978). Anthropology from a Pragmatic Point of View.

[27] Kant I. (1998). Critique of Pure Reason.

[28] Tougas C. (2013). The Phenomena of Awareness Husserl, Cantor, Jung.

[29] Johnston A. (2005). Time Driven Metapsychology and the Splitting of the Drive.

[30] Kant I. (2005). Prolegomena to Any Future Metaphysic.

[31] Brooks R.M. (2011). Un-thought out metaphysics in analytical psychology. J. Anal. Psychol..

[32] Weizman E. (2007). Hollow Land: Israel’s Architecture of Occupation.

[33] Jung C.G. (1973–1975). Letters of C.G. Jung. Volume I: 1906–1950.

[34] Jung C.G. (1970). The Conjunction. The Collected Works of C. G. Jung.

[35] Jung C.G. (1954). On the Nature of the Psyche. The Collected Works of C. G. Jung.

[36] Jung C.G., Adler G., Hull R.F.C. (1975). Letters of C.G. Jung. Volume II: 1951–1961.

[37] Jung C.G. (1938). Psychology and Religion: West and East. The Collected Works of C.G. Jung.

[38] Brooks R.M. (2013). The ethical dimensions of life and analytic work through a Levinasian lens. Int. J. Jungian Stud..

[39] Johnston A. (2008). Žižek’s Ontology A Transcendental Materialist Theory of Subjectivity.

[40] Johnston A. (2004). Against embodiment: The material ground of the immaterial subject. Int. J. Lacanian Stud..

[41] Žižek S. (1989). The Sublime Object of Ideology.

[42] Žižek S., Daly G. (2004). Conversations with Žižek.

[43] Žižek S. (2006). How to Read Lacan.

[44] Zizek S. The matrix, or two sides of perversion. http://www.egs.edu/faculty/slavoj-zizek/articles/the-matrix-or-two-sides-of-perversion/.

[45] Levinas E. (1996). Basic Philosophical Writings.

[46] Levinas E. (1969). Totality and Infinity: An Essay on Exteriority.

[47] Barnard S., Gantt E.E., Williams R.N. (2002). Diachrony, *tuchè,* and the ethical subject in Levinas and Lacan. Psychology for the Other: Levinas Ethics and the Practice of Psychology.

[48] Levinas E. (2008). Otherwise than Being or beyond Essence.

[49] Žižek S. (2006). The Parallax View.

[50] Laplanche J. (2000). The other within: Rethinking psychoanalysis (interview). Radic. Philos..

[51] Laplanche J. (1999). Essays on Otherness.

[52] Scarfone D. (2013). A brief introduction to the work of Jean Laplanche. Int. J. Psychoanal..

[53] Hinton L. (2009). The enigmatic signifier and the decentered subject. J. Anal. Psychol..

[54] Shamdasani S. (2003). Jung and the Making of Modern Psychology the Dream of a Science.

[55] Fordham M. (1975). Memories and thoughts about C.G. Jung. J. Anal. Psychol..

[56] Jung C.G. (1975). Psychology and Religion: West and East. The Collected Works of C. G. Jung.

[57] Jung C.G. (1981). The Psychology of the Child Archetype. The Collected Works of C.G. Jung.

